# Subcutaneous adipose tissue dopamine D2 receptor is increased in prediabetes and T2D

**DOI:** 10.1007/s12020-023-03525-1

**Published:** 2023-09-26

**Authors:** Milica Vranic, Fozia Ahmed, Robin Kristófi, Susanne Hetty, Dariush Mokhtari, Maria K. Svensson, Jan W. Eriksson, Maria J. Pereira

**Affiliations:** 1 Department of Medical Sciences, Clinical Diabetes and Metabolism, Uppsala University, Uppsala, Sweden; 2 Department of Medical Sciences, Renal Medicine, Uppsala University, Uppsala, Sweden

**Keywords:** Dopamine receptors, Dopamine, Adipose tissue, Glucose uptake, Lipolysis, Neuroendocrine

## Abstract

**Purpose:**

To evaluate the dopaminergic signaling in human adipose tissue in the context of obesity and type 2 diabetes (T2D) and potential direct implications in adipose tissue metabolism.

**Methods:**

mRNA and protein expression of dopamine receptors D1 and D2 (DRD1 and DRD2) were determined in subcutaneous adipose tissue from subjects without or with T2D and with different body weight, and correlated with markers of obesity, hyperglycemia, and insulin resistance. Glucose uptake and lipolysis were measured in adipocytes ex vivo following short-term exposure to dopamine, DRD1 receptor agonist (SKF81297), or DRD2 receptor agonist (bromocriptine).

**Results:**

*DRD1* and *DRD2* gene expression in subcutaneous adipose tissue correlated positively with clinical markers of insulin resistance (e.g. HOMA-IR, insulin, and triglycerides) and central obesity in subjects without T2D. Protein expression of DRD2 in subcutaneous adipose tissue, but not DRD1, is higher in subjects with impaired fasting glucose and T2D and correlated positively with hyperglycemia, HbA1c, and glucose AUC, independent of obesity status. DRD1 and DRD2 proteins were mainly expressed in adipocytes, compared to stromal vascular cells. Dopamine and dopaminergic agonists did not affect adipocyte glucose uptake ex vivo, but DRD1 and DRD2 agonist treatment inhibited isoproterenol-stimulated lipolysis.

**Conclusion:**

The results suggest that protein expression of DRD2 in subcutaneous adipose tissue is up-regulated with hyperglycemia and T2D. Whether DRD2 protein levels contribute to T2D development or occur as a secondary compensatory mechanism needs further investigation. Additionally, dopamine receptor agonists inhibit adipocyte beta-adrenergic stimulation of lipolysis, which might contribute to the beneficial effects in lipid metabolism as observed in patients taking bromocriptine.

## Introduction

Dopamine is a neurotransmitter that binds to specific receptors belonging to the G protein-coupled receptor family, which are categorized into two main families: D1-like (D1 and D5) and D2-like (D2, D3, and D4) [1]. These receptors are widely expressed in the central nervous system (CNS) but are also found in the kidney, adrenal glands, gastrointestinal tract, blood vessels, retina, adipose tissue, and heart [2, 3].

Dopamine plays important roles in the CNS, such as motor control, motivation, reward, cognitive function, maternal, and reproductive behaviors [4], as well as in peripheral tissues, as a modulator of cardiovascular function, catecholamine release, hormone secretion, vascular tone, renal blood flow, and gastrointestinal motility [2]. Additionally, dopamine has been shown to play an important role in the regulation of glucose metabolism and energy balance. Studies have shown that dopamine regulates pancreatic endocrine function, including insulin release, in animal models and humans [5]. Moreover, a dopamine receptor D2 (DRD2) agonist, bromocriptine, has recently been approved for the treatment of T2D in the USA, with positive effects on glucose tolerance, insulin sensitivity, and circulating lipids [6–8]. Dopamine is also involved in the modulation of the food reward system, and it has been shown that obese individuals have abnormal brain dopamine activity and reduced striatal DRD2 availability [9, 10]. This may induce pathological overeating to compensate for the decreased activation of reward circuits modulated by dopamine action [9]. Further, it is well known that patients undergoing treatment with antipsychotic drugs, which mainly act as antagonists and/or partial agonists of different dopamine receptors, experience weight gain and related metabolic impairments, such as dyslipidemia, insulin resistance, and the development of T2D [11, 12]. We have recently shown that the atypical antipsychotic drugs, olanzapine, and aripiprazole, act directly on adipocytes inducing gene expression of the energy balance regulating adipokine leptin [13]. In addition, aripiprazole alters adipocyte differentiation and reduces glucose metabolism in differentiated adipocytes. Similarly, we have shown that short-term exposure to second-generation antipsychotics can induce changes in glucose and lipid metabolism in isolated human adipocytes [14]. Whether these effects of the antipsychotic drugs are governed via dopaminergic signaling in adipose tissue is not yet known.

The expression of dopamine receptors in adipose tissue has been confirmed in both rodents and humans [15, 16], and some dopamine actions have been studied [16–19]. However, the current knowledge of dopaminergic signaling in human adipose tissue in the context of obesity and T2D and its direct implications in adipose tissue metabolism is limited.

In this study, we investigated the expression profiles of dopamine receptors in human adipose tissue of subjects with or without obesity and T2D. Furthermore, we studied the potential involvement of dopamine signaling in the regulation of adipocyte glucose and lipid metabolism.

## Materials and methods

### Subjects

**Cohort 1** (*n* = 125) included 95 subjects without T2D (29 men/66 women; body mass index (BMI) 20.2–38.4 kg/m^2^; age 20–72 years) and 30 subjects with T2D (11 men/19 women; BMI 22.4–40.1 kg/m^2^; age 31–71 years), including 12 patients (3 men/9 women; age: 31–61 years, BMI: 31.1–40.1 kg/m^2^) with obesity and T2D undergoing Roux-en-Y Gastric Bypass (RYGB). From all subjects abdominal subcutaneous adipose tissue (SAT) was obtained by needle biopsies from the lower part of the abdomen after local dermal anesthesia with lidocaine (Xylocaine, AstraZeneca). All subjects were recruited at the Uppsala University Hospital.

The subjects with T2D were on a stable dose of metformin as their only antidiabetic medication. Sex, age, and BMI-matched patients without T2D [20] and patients undergoing RYGB [21, 22] have previously been described in detail. In patients undergoing RYGB SAT obtained by needle biopsies were available at several time points after surgery (at 4, 24, and 104 weeks).

**Cohort 2** (*n* = 54) included 39 healthy kidney donors (21 men/18 women, age: 30–71 years, BMI: 20.0–31.7 kg/m^2^) and 15 subjects undergoing bariatric surgery without T2D (4 men/11 women, age: 19–63 year, BMI: 31.3–58.2 kg/m^2^) in which paired samples of SAT and omental adipose tissue (OAT) were obtained during surgery at Uppsala University Hospital or the Sahlgrenska University Hospital. These samples were used to investigate the DRD1 and DRD2 gene and protein expression.

Subjects with type 1 diabetes, endocrine disorders, cancer, or other major diseases, as well as subjects taking medications such as antipsychotics, antidepressants, neuroleptic drugs, or dopaminergic drugs, were excluded from the study. Blood samples were collected after overnight fasting ( > 10 h) for the analysis of HbA1c, plasma glucose and lipids, and serum insulin at the Department of Clinical Chemistry at Uppsala University Hospital or Sahlgrenska University Hospital using standard clinical methods. Anthropometric and clinical characteristics of all subjects in the two cohorts are shown in Table 1.

**Table 1:** Subject characteristics

Variable	Subject with needle biopsies (cohort 1) (*n* = 125)		Surgical biopsies (cohort 2) (*n* = 54)
	Without T2D	T2D	Without T2D
Men/Women (number)	29/66	11/19	25/29
Age (years)	50 ± 18	54 ± 9	48 ± 12
BMI (kg/m^2^)	27.1 ± 3.9	32.9 ± 5.2***	31.2 ± 10.7
Waist-hip-ratio	0.89 ± 0.09	0.99 ± 0.06***	0.92 ± 0.10
Plasma glucose (mmol/L)	5.8 ± 0.7	8.2 ± 1.6***	5.5 ± 0.6
Serum insulin (mU/L)	8.8 ± 4	22.2 ± 12.4***	12.5 ± 7.5
HbA1c, IFCC (mmol/mol)	34.9 ± 3.9	51.1 ± 9.8***	34.6 ± 3.7
HOMA IR	2.3 ± 1.2	7.9 ± 4.9***	3.1 ± 2.3
Plasma total cholesterol (mmol/L)	5.2 ± 1.1	4.8 ± 0.9	4.8 ± 1
Plasma HDL-cholesterol (mmol/L)	1.3 ± 0.4	1.1 ± 0.2**	1.4 ± 0.4
Plasma LDL-cholesterol (mmol/L)	2.8 ± 1.1	3.1 ± 0.8	3.1 ± 0.9
Plasma triglycerides (mmol/L)	1.5 ± 1	1.8 ± 0.8	1.3 ± 0.6

Data are median ± SD **p < 0.01; ***p < 0.001, compared to subjects without T2D

*BMI* body mass index, *HbA1c* glycated hemoglobin, *HOMA IR* homeostatic model assessment of insulin resistance index (fasting blood glucose * fasting insulin / 22.5), *LDL* low-density lipoprotein, *HDL* high-density lipoprotein

Adipose tissue samples were snap-frozen in liquid nitrogen and used for mRNA and protein expression analyses. Furthermore, mature adipocytes were isolated from adipose tissue and used for adipocyte size measurements and ex vivo analyses of lipolysis and glucose uptake without and with dopamine and dopamine receptors agonists. Importantly, not all assessments were done for all subjects due to limitations in the amount of adipose tissue available.

### Adipocyte glucose uptake and lipolysis, and cell size

Mature adipocytes were isolated from SAT (cohort 1) and used for the assessment of ex vivo lipolysis (*n* = 16), glucose uptake (*n* = 46), and adipocyte size measurement (n = 63) as previously reported [20, 23–25]. Briefly, the tissue was digested in collagenase solution (1.0 mg/mL, from *Clostridium histolyticum*, Roche) in Hank’s medium (Medium 199, Gibco, Life Technologies) supplemented with 5.6 mM glucose, 4% bovine serum albumin (BSA, Sigma), 150 nM adenosine (Sigma), pH 7.4, in a shaking water bath at 37 °C and 105 RPM. Following digestion with collagenase, the tissue was filtered through a nylon mesh, and mature adipocytes were isolated, washed, and suspended in Hank’s medium. The cell diameter of 100 consecutive cells was measured by light microscopy, as previously reported [23]. Lipolysis was determined by measuring the levels of glycerol in the medium released by adipocytes, and glucose uptake was measured by the adipocyte ^14^C glucose uptake into adipocytes, following the incubation without or with insulin (25 and 1000 µU/mL).

### Impact of dopamine and dopamine receptor agonists on adipocyte glucose uptake and lipolysis

To investigate the effect of dopamine on adipocyte glucose uptake and lipolysis, isolated adipocytes (cohort 1) were incubated for 30 min with either physiological (1 and 50 nM) or supra-physiological concentrations of dopamine (100 nM) [26, 27] before glucose uptake (*n* = 7) or lipolysis (*n* = 3) assays, as previously reported [20, 25]. The supra-physiological concentration of dopamine was used to ensure complete saturation of the receptors. Additionally, to investigate the involvement of the dopamine receptors, adipocytes were also treated with the DRD1 receptor agonist SKF81297 (10 nM; pKi=8.7), or DRD2 receptor agonist bromocriptine (100 nM; pKi=7.3–8.3) [28], prior to glucose uptake (*n* = 3) or lipolysis (*n* = 4) assays.

### Gene expression

SAT (cohorts 1 and 2, *n* = 168) and OAT (cohort 2, *n* = 52) were used for total RNA isolation using the RNeasy lipid tissue mini kit (Qiagen). The concentration and purity assessment of total RNA was performed with the Nanodrop (Thermo Scientific). RNA samples were used to synthesize cDNA using a High-Capacity cDNA Reverse Transcriptase kit (Applied Biosystems) according to the manufacturer’s guidelines. DNA contamination was monitored by including no reverse transcriptase (NRT) controls in the cDNA synthesis reactions for a sub-set of adipose tissue samples. Expression levels of *DRD1* (Hs00265245), *DRD2* (Hs00241436), and *DRD4* (Hs00609526) were determined using TaqMan gene expression assays (Thermo Fisher), detected using the QuantStudio 3 sequence detection system (Applied Biosystem) and calculated as 2^-deltaCT^. The gene expression levels were normalized to the housekeeping gene *GUSB* (Hs00939627).

### Western blot

Total protein lysates were prepared from SAT and OAT (cohorts 1 and 2, *n* = 27/8 for SAT/OAT), as previously reported [29]. Briefly, adipose tissue was homogenized with lysis buffer (25 mM Tris-HCl; 0.5 mM EGTA; 25 mM NaCl; 1% Nonidet P-40; 1 mM Na3VO4; 10 mM NaF (all from Sigma); 100 nM okadaic acid (Alexis Biochemicals), 1X Complete protease inhibitor cocktail (Roche), and pH 7.4), and samples were rocked for 60 min at 4 °C and centrifuged at 12,000 × *g* for 15 min at 4 °C. The lysate was collected, and the protein concentration was determined using a BCA protein assay kit (Pierce, Thermo Scientific). Protein lysates (10–15 µg) were separated by SDS-PAGE (5–8% gradient, BioRad), transferred to nitrocellulose membranes, and blocked for one hour at room temperature with 0.05% tween-phosphate buffer saline (PBST, Medicago) with 5% BSA. Membranes were incubated overnight in the solution of the primary antibodies: anti-DRD1 (1:1000; Sigma Aldrich D2944) and anti-DRD2 (1:1000; Merck Millipore AB5084P), previously validated [30]. Stain-free blot imaging was used to quantify the total protein for each sample and to normalize DRD1 and DRD2 protein levels [31]. Membranes were then washed with PBST buffer and incubated with appropriate horseradish peroxidase-conjugated anti-rabbit and anti-rat (Cell Signaling Technologies) secondary antibodies. Protein bands were visualized using enhanced chemiluminescence with a high-resolution field and quantified with ChemiDocTM MP System (BioRad).

### Statistical analysis

Descriptive data are presented as mean ± SEM unless stated otherwise. All data were first checked for normality using the Shapiro-Wilk test and by analyzing histograms. Spearman’s correlation test was performed between *DRD1* mRNA, DRD1, and DRD2 protein expression and clinical markers of obesity, hyperglycemia, and insulin resistance. Kendall’s tau-b (*τ*_b_) was used for *DRD2* mRNA expression correlation analysis to avoid potential problems of non-normality, sensitivity to outliers, and ranked expression values [32]. Samples without detectable levels of *DRD2* mRNA were set to 1/10 of the lowest detection level. Mann–Whitney U or two-sample t-test was used for analyses between 2 independent groups. Protein data were log-transformed prior to analyses. Comparison between more than 2 groups was made using one-way ANOVA. The false discovery rate with the original Benjamini and Hochberg method was used for multiple comparison corrections. All statistical analyses were performed using IBM SPSS Statistics 28 and GraphPad Prism 9.5 software, and the *p* < 0.05 value was considered statistically significant.

## Results

### DRD1 and DRD2 gene and protein expression levels in human adipose tissue (SAT vs OAT)

**Fig. 1:**
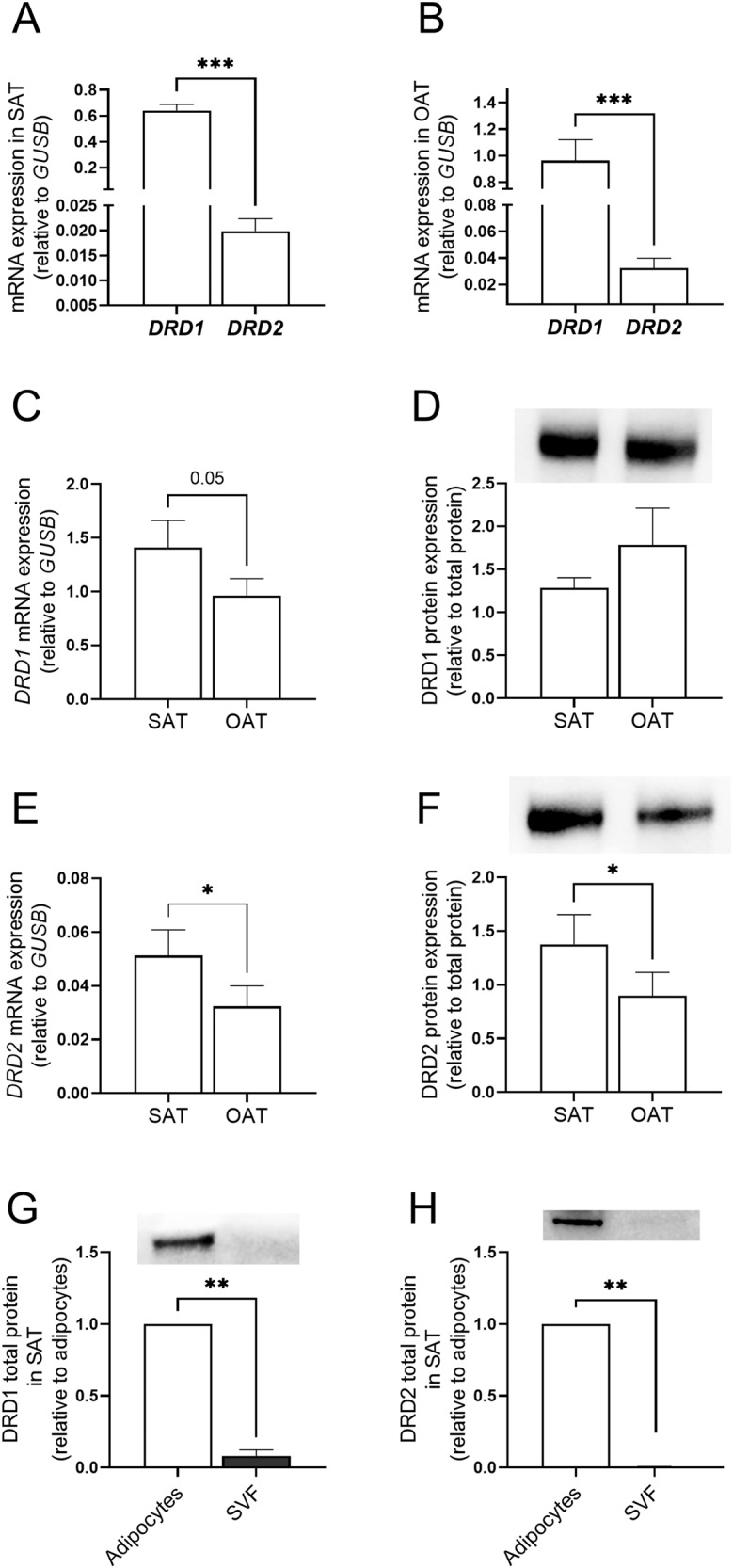
Dopamine receptors expression in human adipose tissue. **A** *DRD1* and *DRD2* mRNA expression in human SAT. *n* = 87, subjects without T2D, cohort 1. **B** *DRD1* and *DRD2* mRNA expression in human OAT. *n* = 52, cohort 2. **C**, **E** *DRD1* and *DRD2* mRNA expression in paired SAT and OAT. *n* = 52, cohort 2. **D**, **F** DRD1 and DRD2 protein expression in paired SAT and OAT. *n* = 7, cohort 2. **G**, **H** DRD1 and DRD2 total protein expression in mature adipocytes and SVF in SAT. *n* = 3, subjects without T2D, cohort 1. Western blot data are shown as relative to total protein and a representative immunoblot of total protein levels is provided in online resource 1. Data are shown as mean ± SEM. *p < 0.05, **p < 0.01, ***p < 0.001. SAT subcutaneous adipose tissue, OAT omental adipose tissue, SVF stromal vascular fraction

mRNA expression of *DRD1* was detected in all samples, whilst *DRD2* expression was detected in ~85% of SAT and ~80% of OAT samples. Furthermore, the mRNA expression of *DRD1* was significantly higher than *DRD2* expression in both SAT and OAT (*p* < 0.001) (Fig. 1A, B). *DRD4* mRNA expression was also measured in human SAT and OAT but was not detected in these tissues.

*DRD1* and *DRD2* mRNA expression was measured in paired samples of SAT and OAT obtained from subjects without T2D (cohort 2). *DRD1* mRNA expression was ~30% lower in OAT compared to SAT (*p* = 0.05) (Fig. 1C), but the protein levels did not differ between the depots (Fig. 1D). *DRD2* mRNA and protein expression were ~40% lower in OAT compared to SAT (*p* < 0.05) (Fig. 1E, F, respectively).

Additionally, we assessed protein levels of DRD1 and DRD2 in adipocytes and the stromal vascular fraction (SVF) obtained from SAT. To account for different proportions of adipocytes and SVF cells in SAT, we calculated the total protein proportion of adipocytes and SVF in the whole-adipose tissue sample and determined that SVF contributed with less than 1% for both DRD1 and DRD2 protein expression (*p* < 0.01) (Fig. 1G, H).

### DRD1 and DRD2 gene and protein expression in SAT from subjects with and without T2D and obesity

**Fig. 2:**
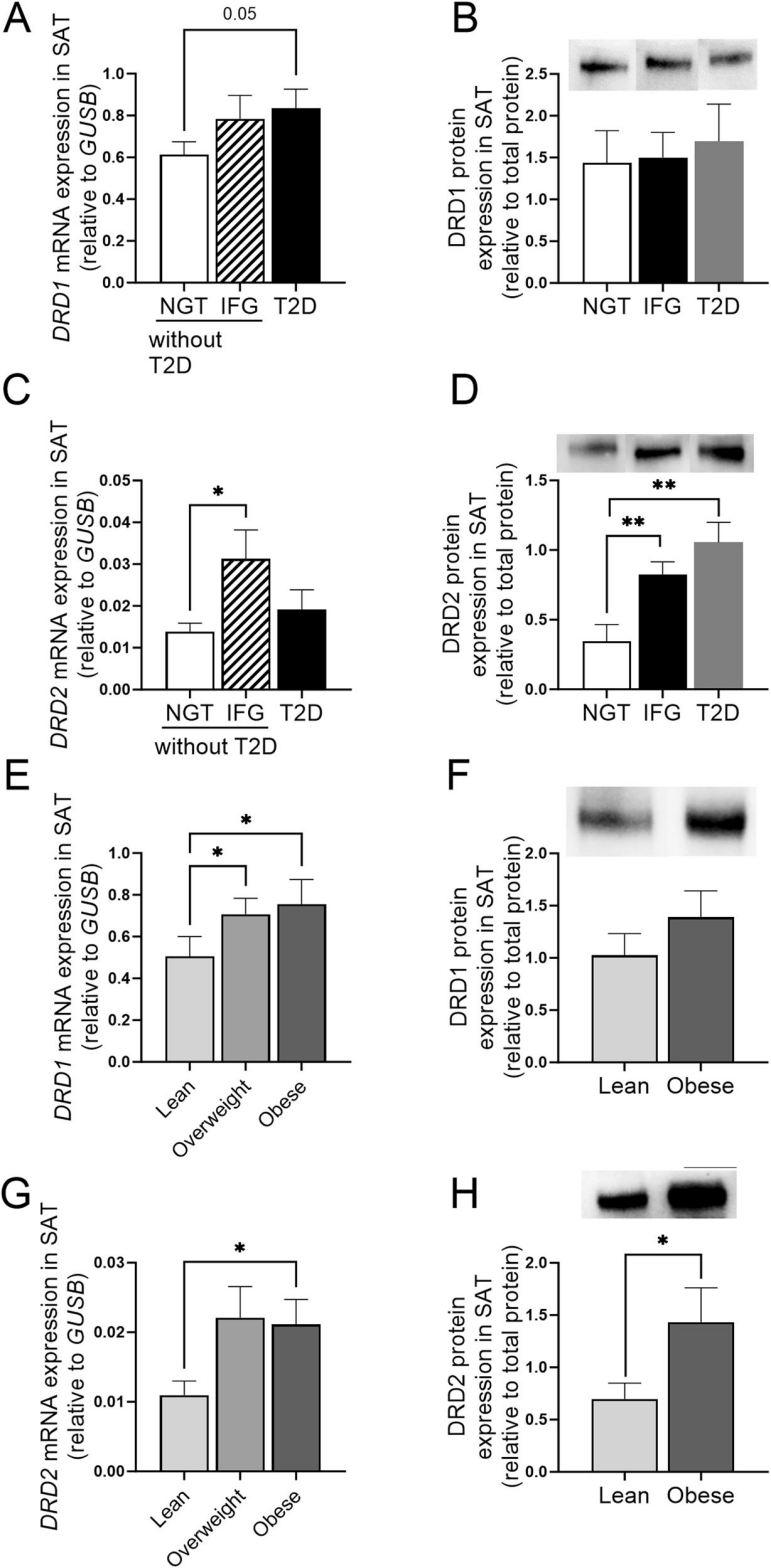
Dopamine receptors expression in SAT from subjects with and without T2D and obesity. **A**, **C** *DRD1* and *DRD2* mRNA expression in SAT of subjects with NGT, IFG, and T2D, *n* = 64/23/29, NGT/IFG/T2D from cohort 1. **B**, **D** DRD1 and DRD2 protein expression in SAT of subjects with NGT, IFG, and T2D from cohort 1, *n* = 4/4/7. **E**, **G** *DRD1* and *DRD2* gene expression in lean, overweight, and subjects with obesity. *n* = 25/44/18 from cohort 1. **F**, **H** DRD1 and DRD2 protein expression in SAT of lean subjects and subjects with obesity. *n* = 5/6 from cohort 1. Western blot data are shown as relative to total protein and a representative immunoblot of total protein is provided in online resource 1. Data are shown as mean ± SEM. *p < 0.05, **p < 0.01. SAT subcutaneous adipose tissue, NGT normal glucose tolerance, IFG impaired fasting glucose, T2D type 2 diabetes

Gene expression of *DRD1* and *DRD2* was measured in SAT from subjects without and with T2D (cohort 1). Subjects without T2D were further subdivided into those with normal glucose tolerance (NGT) or impaired fasting glucose (IFG) using the WHO criteria of IFG with a lower cut-off of 6.1 mmol/L [33]. *DRD1* mRNA expression in SAT was significantly increased in subjects with T2D compared to subjects without T2D with NGT but not in subjects without T2D with IFG (Fig. 2A). No differences in protein expression were found between the groups (Fig. 2B). For *DRD2*, the mRNA levels were significantly higher in subjects without T2D with IFG compared to those with NGT but no significant difference was found between subjects with T2D and those with NGT (Fig. 2C). However, DRD2 protein was significantly increased by about 2-fold in subjects with IFG and those with T2D, compared to subjects with NGT (Fig. 2D).

Next, we investigated whether DRD1 and DRD2 gene and protein expression in SAT differed between lean, overweight, and obese subjects without T2D. We found that *DRD1* and *DRD2* gene expression in SAT was up-regulated in subjects with overweight (BMI: 25–29 kg/m^2^) or subjects with obesity (BMI: ≥30 kg/m^2^), compared to lean subjects (Fig. 2E, G). Subjects with obesity had ~ 2-fold higher protein expression of DRD2 than lean subjects (Fig. 2H), but DRD1 protein levels only showed a tendency to higher levels in subjects with obesity compared to lean subjects (Fig. 2F).

### Impact of weight loss on DRD1 and DRD2 expression in SAT from subjects with obesity and T2D

**Fig. 3:**
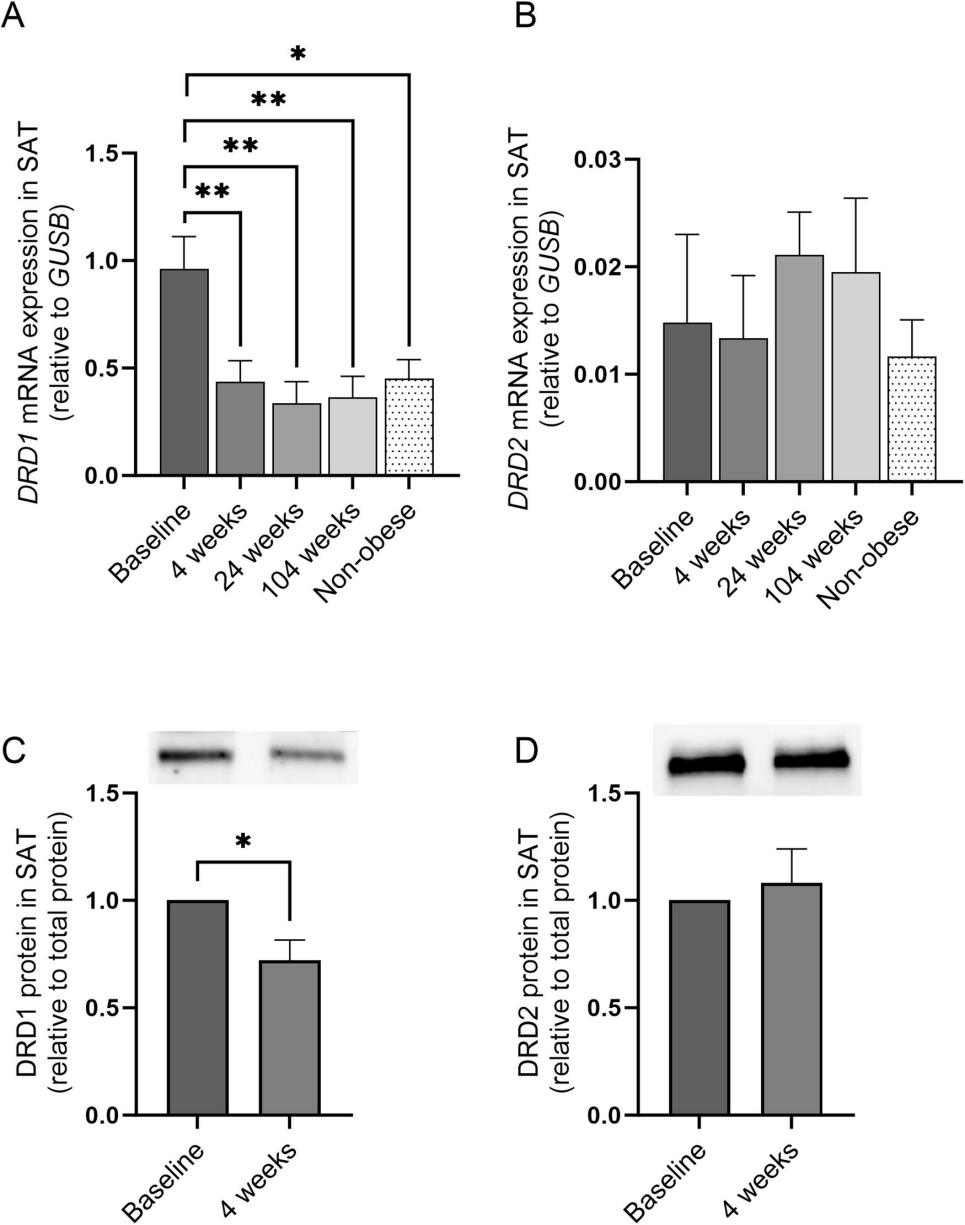
Impact of weight loss with bariatric surgery on DRD1 and DRD2 expression in SAT from subjects with obesity and T2D. **A**, **B** *DRD1* and *DRD2* mRNA expression in subjects with obesity and T2D at baseline and 4, 24, and 104 weeks post-RYGB, and in controls without obesity and T2D. *n* = 8–12 from cohort 1. **C**, **D** DRD1 and DRD2 protein expression in subjects with obesity and T2D at baseline and 4 weeks post-RYGB. *n* = 7–9 from cohort 1. Western blot data are shown as relative to total protein and a representative immunoblot of total protein levels is provided in online resource 1. Data are shown as mean ± SEM. *p < 0.05, **p < 0.01. SAT subcutaneous adipose tissue

To determine the impact of weight loss, gene and protein expression of DRD1 and DRD2 was also measured in SAT from subjects with obesity and T2D, before (baseline) and at several time points after the RYGB surgery (at 4, 24, and 104 weeks post-RYGB; cohort 1) (Fig. 3). A rapid decrease was observed in the *DRD1* gene and protein expression levels 4 weeks post-RYGB (*p* < 0.05) (Fig. 3A, C), with sustained mRNA levels up to 104 weeks post-surgery (*p* < 0.01) (Fig. 3A). There was no difference in the *DRD2* gene and protein expression levels before and after RYGB (Fig. 3B, D). In addition, these subjects were compared to subjects without T2D (age- and BMI-matched subjects at 104 weeks post-RYGB) that did not undergo weight-loss surgery (“non-obese” controls, Fig. 3). *DRD1* gene expression levels at all time points post-RYGB were similar to levels in the BMI- and age-matched “non-obese” control group (Fig. 3A).

### Correlation of *DRD1* and *DRD2* gene expression in SAT with insulin resistance and obesity

*DRD1* and *DRD2* gene expression in human SAT correlated with hyperglycemia, insulin resistance, and obesity when considering both subjects with and without T2D (cohort 1), and results are shown in Tables 2 and 3. *DRD1* gene expression in SAT correlated positively with BMI, WHR (*p* < 0.05, both), and adipocyte size (p < 0.001); and fasting glucose (*p* < 0.05), HOMA-IR, insulin, and fasting triglycerides (*p* < 0.001, all) (Table 2). When dividing subjects by T2D status (with and without), similar correlations were observed in subjects without T2D, but in the T2D group, *DRD1* gene expression only correlated positively with adipocyte size (*p* < 0.05).

**Table 2:** *DRD1* gene expression correlations with markers of obesity and insulin resistance

	*DRD1* mRNA in SAT					
	*All (n* = *116)*		*Without T2D (n* = *87)*		*T2D (n* = *29)*	
	*Rho*	*p value*	*Rho*	*p value*	*Rho*	*p value*
BMI	**0.273**	**0.003**	**0.270**	**0.011**	0.151	0.435
WHR	**0.193**	**0.039**	**0.233**	**0.031**	−0.175	0.363
Fasting triglycerides	**0.437**	**<0.001**	**0.389**	**<0.001**	0.266	0.163
Fasting glucose	**0.206**	**0.027**	0.076	0.485	*0.325*	*0.085*
Fasting insulin	**0.354**	**<0.001**	**0.323**	**0.002**	0.264	0.166
HbA1c	*0.161*	*0.085*	0.029	0.792	0.241	0.208
HOMA-IR	**0.373**	**<0.001**	**0.330**	**0.002**	0.265	0.165
^a^Ex vivo basal glucose uptake	−0.072	0.564	−0.141	0.328	0.157	0.548
^a^Ex vivo insulin-stimulated glucose uptake	−0.120	0.373	−0.182	0.261	−0.172	0.510
^a^Ex vivo maximal insulin-stimulated glucose uptake	−0.117	0.348	−0.121	0.403	−0.191	0.462
^b^Ex vivo basal lipolysis	*0.280*	*0.093*	0.268	0.267	0.280	0.261
^b^Ex vivo ISO-stimulated lipolysis fold change	−0.150	0.375	0.253	0.297	*−0.430*	*0.075*
^c^Subcutaneous adipocyte size	**0.427**	**<0.001**	**0.413**	**<0.001**	**0.503**	**0.034**

Table presents Spearman´s Rho correlation coefficient. Significant Spearman correlation values are bolded, *p* < 0.05, and italics, *p* < 0.1

SAT was obtained from cohort 1

*BMI* body mass index, *WHR* waist-to-hip ratio, *HbA1c* glycated glucose, *HOMA-IR* homeostatic model assessment for insulin resistance, *ISO* isoproterenol

^a^*n* = 67/50/17; ^b^*n* = 37/19/18; ^c^*n* = 87/69/18 for all/without T2D/T2D

**Table 3:** *DRD2* gene expression correlations with markers of obesity and insulin resistance

	*DRD2* mRNA in SAT					
	*All (n* = *116)*		*Without T2D (n* = *87)*		*T2D (n* = *29)*	
	*τ*_b_	*p value*	*τ*_b_	*p value*	*τ*_b_	*p value*
BMI	0.029	0.652	*0.127*	*0.085*	−0.141	0.291
WHR	0.097	0.131	**0.169**	**0.023**	0.086	0.521
Fasting triglycerides	0.052	0.417	*0.143*	*0.053*	−0.207	0.122
Fasting glucose	0.103	0.113	**0.245**	**0.001**	−0.059	0.664
Fasting insulin	**0.127**	**0.047**	**0.268**	**0.000**	−0.109	0.417
HbA1c	−0.007	0.918	0.077	0.311	−0.130	0.343
HOMA-IR	**0.127**	**0.047**	**0.267**	**0.000**	−0.152	0.258
^a^Ex vivo basal glucose uptake	0.038	0.657	−0.033	0.738	0.264	0.146
^a^Ex vivo insulin-stimulated glucose uptake	0.025	0.788	−0.060	0.591	*0.355*	*0.051*
^a^Ex vivo maximal insulin-stimulated glucose uptake	−0.105	0.211	*−0.180*	*0.068*	−0.013	0.939
^b^Ex vivo basal lipolysis	0.099	0.393	0.077	0.648	0.174	0.321
^b^Ex vivo maximal ISO-stimulated lipolysis	0.005	0.969	−0.053	0.752	0.000	1.000
^c^Subcutaneous adipocyte size	*0.137*	*0.063*	*0.162*	*0.052*	0.080	0.647

Table presents Kendall’s tau-b (*τ*_b_) correlation coefficient. Significant correlation values are in bold, *p* < 0.05, and italics, *p* < 0.1

SAT was obtained from cohort 1

*BMI* body mass index, *WHR* waist-to-hip ratio, *HbA1c* glycated glucose, *HOMA-IR* homeostatic model assessment for insulin resistance, *ISO* isoproterenol

^a^*n* = 59/46/13; ^b^*n* = 30/16/14; ^c^*n* = 77/63/14 for all/control/T2D

In subjects without and with T2D taken together, *DRD2* gene expression in SAT correlated positively with fasting insulin and HOMA-IR (*p* < 0.05, both) (Table 3). In subjects without T2D, similar correlations were observed, with the addition of a positive correlation with WHR (*p* < 0.05) and fasting glucose (*p* = 0.001). In subjects with T2D, *DRD2* expression tended to correlate positively with ex vivo insulin-stimulated glucose uptake (*p* = 0.051).

### Correlation of DRD1 and DRD2 protein expression with hyperglycemia and obesity

**Fig. 4:**
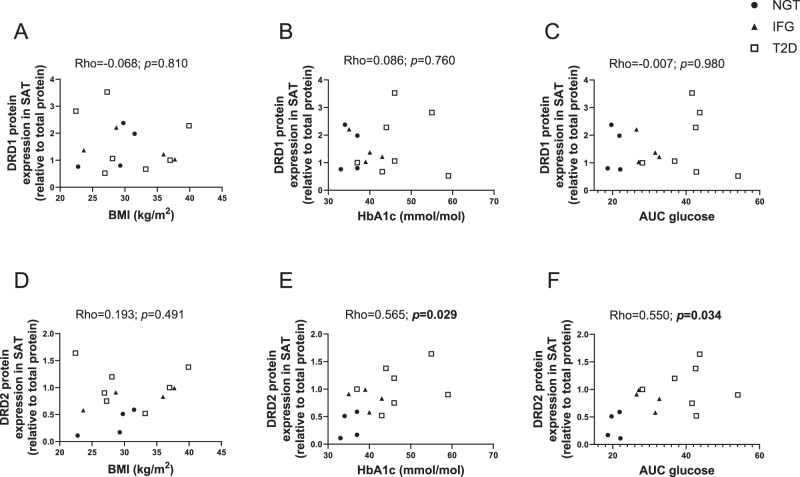
DRD1 and DRD2 protein expression correlation with markers of obesity and hyperglycemia. **A**–**C** DRD1 and **D**–**F** DRD2 protein expression association with BMI, HbA1c, and AUC glucose during an OGTT. Dopamine receptors expression in SAT from subjects with and without T2D and obesity. *n* = 4/4/7 for NGT/IFG/T2D from cohort 1. Significant Spearman correlation values are bolded, *p* < 0.05. NGT normal glucose tolerance, IFG impaired fasting glucose, T2D type 2 diabetes, BMI body mass index, HbA1c glycated hemoglobin

Since protein levels of DRD1 and DRD2 generally did not follow mRNA expression patterns, we performed correlation analyses between DRD1 and DRD2 protein expression and obesity and hyperglycemia in subjects without T2D and with NGT or IFG, and T2D (cohort 1), respectively (Fig. 4). When considering both subjects without and with T2D, DRD2 protein expression correlated positively with HbA1c (*p* = 0.029) and AUC glucose during OGTT (*p* = 0.034), independently of obesity status (Fig. 4E, F, respectively, and Table 4). DRD2 protein expression was not significantly associated with the insulin resistance marker HOMA-IR. No significant correlations were observed between DRD1 protein expression and the clinical characteristics of the subjects (Fig. 4A–C).

**Table 4:** Multiple regression analysis of DRD1 and DRD2 protein expression in SAT

DRD1 protein in SAT					
	Model 1	Model 2	Model 3	Model 4	Model 5
* R*^2^	0.021	0.024	0.017	0.018	0.038
Model *p* value	0.879	0.864	0.901	0.894	0.792

Variable	*β;* p	*β;* p	*β;* p	*β;* p	*β;* p
BMI	−0.126; 0.674	−0.133; 0.651	—	—	−0.146; 0.616
T2D	—	—	0.145; 0.719	0.182; 0.710	0.141; 0.629
HbA1c	0.053; 0.858	—	−0.021; 0.958	—	—
AUC glucose	—	0.075; 0.798	—	−0.065; 0.894	—

DRD2 protein in SAT					
	Model 1	Model 2	Model 3	Model 4	Model 5
* R*^2^	0.470	0.390	0.412	0.373	0.304
Model *p* value	**0.022**	*0.052*	**0.041**	*0.061*	*0.066*

Variable	*β;* p	*β;* p	*β;* p	*β;* p	*β;* p
BMI	0.348; 0.130	0.245; 0.298	—	—	**0.537; 0.025**
T2D	—	—	0.333; 0.295	0.346; 0.382	0.179; 0.420
HbA1c	**0.663; 0.009**	—	0.366; 0.251	—	—
AUC glucose	—	**0.586; 0.023**	—	0.298; 0.450	—

SAT samples are from cohort 1 (*n* = 15). Values are bolded, *p* < 0.05, and italics, *p* < 0.1

*β* standardised beta coefficient, *BMI* body mass index, *HbA1c* glycated glucose

### Effects of dopamine and dopamine receptor agonists on adipocyte glucose uptake and lipolysis

**Fig. 5:**
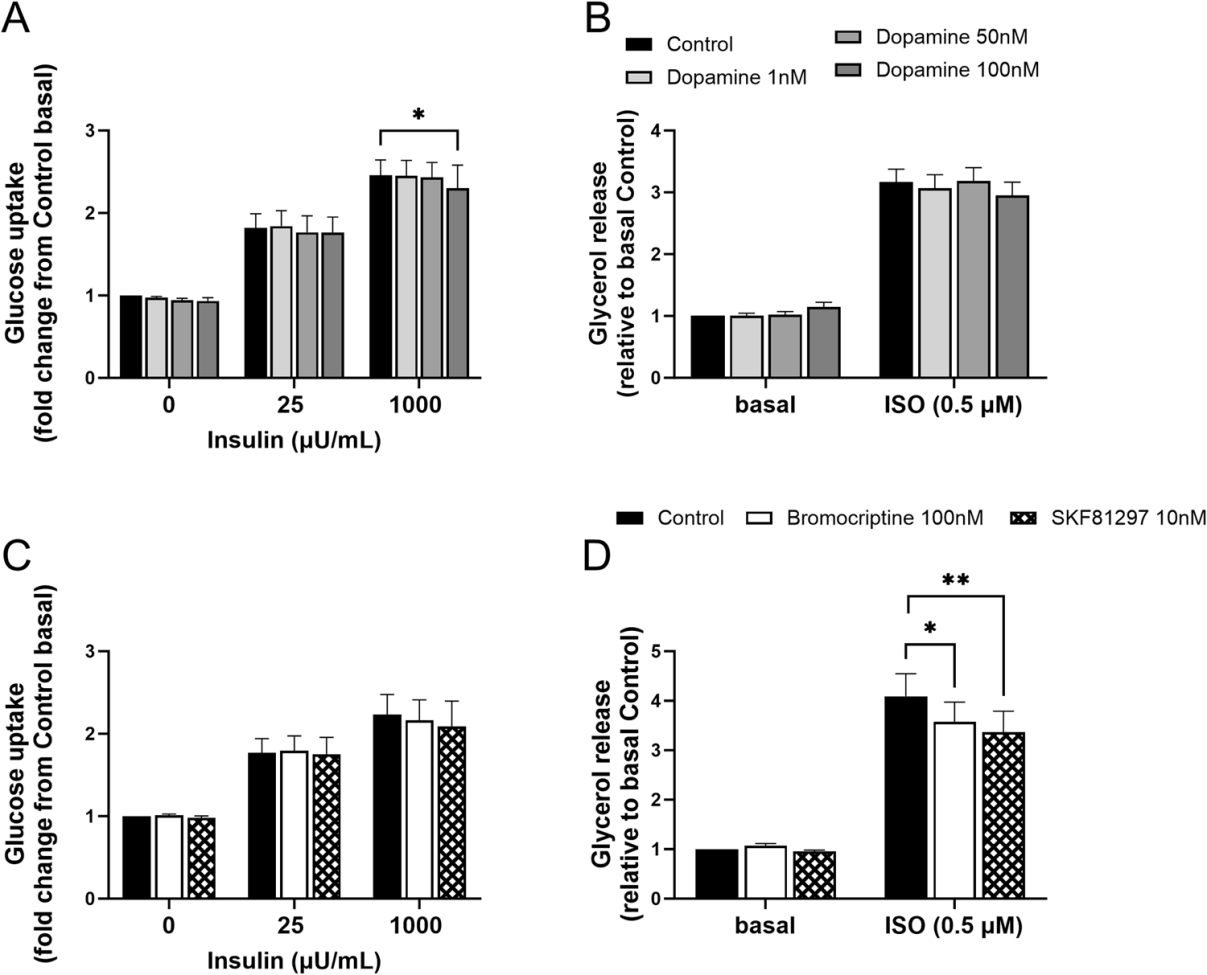
Effects of dopamine and dopamine receptor agonists on subcutaneous adipocyte metabolism. Dopamine effects on (**A**) basal and insulin-stimulated adipocyte glucose uptake and on (**B**) basal and isoproterenol-stimulated adipocyte lipolysis. (*n* = 7 and 3 without T2D from cohort 1, respectively). SKF81297 (D1 receptor agonist) and bromocriptine (D2 receptor agonist) effects on (**C**) basal and insulin-stimulated adipocyte glucose uptake, and (**D**) basal and isoproterenol-stimulated adipocyte lipolysis (*n* = 3 and 4 without T2D from cohort 1, respectively). Data are shown as mean ± SEM. *p < 0.05, **p < 0.01. ISO isoproterenol

Dopamine at physiological (1 and 50 nM) concentrations did not alter adipocyte basal or insulin-stimulated glucose uptake ex vivo, while a supra-physiological concentration (100 nM) caused a minor reduction in insulin-stimulated glucose uptake by ~10% (*p* < 0.05) (Fig. 5A). Furthermore, dopamine did not affect either basal or isoproterenol-stimulated lipolysis in adipocytes, ex vivo (Fig. 5B).

DRD1 receptor agonist (SKF81297) and DRD2 receptor agonist (bromocriptine) did not affect basal or insulin-stimulated glucose uptake (Fig. 5C). However, while basal lipolysis was not affected by either receptor agonist, SKF81297 and bromocriptine treatment inhibited isoproterenol stimulation of lipolysis by ~12% (*p* < 0.05) and ~17% (*p* < 0.01), respectively (Fig. 5D).

## Discussion

Taken together, the results in this study suggest that DRD2 protein expression is increased in subjects with obesity, impaired fasting glucose (IFG), and type 2 diabetes (T2D), and positively correlated with hyperglycemia. Additionally, DRD1 protein expression is increased in subjects with overweight/obesity, but not T2D. Further, adipocyte isoproterenol-stimulated lipolysis was reduced following treatment with DRD1 and DRD2 agonists.

Adipose tissue is an important endocrine organ involved in the regulation of whole-body energy homeostasis and has a crucial role in linking obesity to increased risk of insulin resistance, dyslipidemia, and T2D [34]. Literature shows that dopamine affects whole-body energy balance by regulating eating-related behaviors in the CNS and insulin secretion from the pancreas [35, 36]. Despite growing evidence for peripheral dopamine production and action, and expression of dopamine receptors in peripheral tissues, including adipose tissue, most studies up until now have focused on dopamine functions in the CNS.

In this study, we confirm that human adipose tissue expresses *DRD1* and *DRD2*, with a significantly higher expression of *DRD1* compared to *DRD2*, in both SAT and OAT. Previous reports have also shown the expression of *DRD4* in human cell lines and visceral adipose tissue [15, 16], but we have not detected the expression of *DRD4* in SAT or OAT in this study. Apart from cell- and depot-specific differences in *DRD4* expression, discrepant findings could also be due to the use of methods with different detection sensitivity (TaqMan vs. high throughput chip platform).

The gene expression of *DRD1* was lower in SAT compared to OAT, but this was not found for the protein expression. As far as we know, a direct comparison of the *DRD1* expression between SAT and OAT has not previously been performed. A previous publication [37] demonstrated that *DRD2* gene expression was increased in visceral (omental) compared to subcutaneous adipocytes. In contrast, we show that the DRD2 gene and protein expression was lower in OAT compared to SAT. These conflicting findings could be due to differences in the subjects‘ characteristics and populations studied in the different studies. To account for different proportions of the receptors on adipocytes and SVF cells in SAT, we determined the relative protein levels for these fractions, but DRD1 and DRD2 protein expression were mainly found in adipocytes when compared to SVF and this is in accordance with previously published data [37].

We observed a significant increase in DRD2 receptor protein levels even prior to the development of T2D, in subjects with IFG and obesity. Accordingly, DRD2 protein expression positively correlated with hyperglycemia markers. This led us to hypothesize that either the DRD2 receptors on adipocytes could have implications for the development of insulin resistance and T2D, or their expression could be changed as a compensatory mechanism counterbalancing for example hyperglycemia or altered nutrient availability. Future studies should investigate how the exposure of adipose tissue to different glucose concentrations affects the expression of DRD2. Adipocyte exposure to varying glucose concentrations and measuring levels of DRD2 could provide a response regarding the direction of the changes. Further, longitudinal studies investigating the impact of T2D development on DRD2 levels could be useful to overcome the limitation of our cross-sectional study and produce a clearer picture of the role of DRDs´ in adipose tissue functions. Previously, it has been reported that the *DRD2* gene expression does not change in OAT of subjects with obesity and T2D, compared to subjects with obesity but without T2D [16]. The apparent discrepancy could be due to the type of adipose tissue studied, while we studied SAT, the previous group investigated OAT, and also due to the differences in the studied populations.

*DRD1* gene expression was increased in subjects with obesity with a tendency to be additionally increased with T2D and positively correlated with adiposity markers and insulin resistance. However, the DRD1 protein levels did not follow the gene expression data, when comparing subjects without and with obesity or T2D. Additionally, no correlations were found between DRD1 protein expression and markers of insulin resistance and obesity. Thus, the results of the association study should be considered with reservation. The significantly discordant changes between the protein and mRNA levels in our study suggest that post-transcriptional or post-translational regulatory mechanisms regulate the protein expression of DRD1 [38]. This highlights the necessity to assess both mRNA and protein levels of dopamine receptors in future experimental designs. Interestingly, DRD1 gene and protein expression was markedly decreased shortly after RYGB surgery, and mRNA levels were sustained during follow-up for up to 104 weeks. RYGB is an effective obesity treatment, and patients experience rapid changes in weight and improvements in glycemic control and insulin sensitivity [22]. There is also evidence that RYGB can modulate dopamine receptors and dopamine action levels in the brain and periphery (e.g. pancreas) independently of weight loss [39–41]. Whether observed changes in adipose tissue *DRD1* expression are governed by weight loss or other mechanisms, potentially through communication with other organs, requires further investigation.

It has previously been shown that bromocriptine, a DRD2 receptor agonist, has positive effects on glucose tolerance, insulin sensitivity, and cardiovascular risk in subjects with and without T2D [6, 42, 43] and is approved as a treatment for T2D in the USA by the FDA [44]. The glucose metabolic effects of bromocriptine, have mainly been attributed to its actions in the CNS [8]. However, with the known expression of dopamine receptors outside the CNS, the potential direct effects of dopamine on adipocyte metabolism are possible. Our functional assessment demonstrated that dopamine, SKF38393 (DRD1 agonist), and bromocriptine (DRD2 agonist) with concentrations up to 100 nM do not directly affect subcutaneous adipocyte glucose uptake ex vivo. A recent study showed that high concentrations (10 µM) of dopamine or bromocriptine can act to enhance insulin signaling in rat mesenchymal adipose tissue [45]. The apparent discrepant findings could be due to differences in species, adipocyte origin, or the higher concentrations used in the abovementioned study.

Additionally, our results show that acute ex vivo treatment of subcutaneous adipocytes with dopamine does not directly affect lipolysis, however, treatment with DRD1 and DRD2 agonists inhibited isoproterenol-stimulated lipolysis. These results are consistent with previous studies showing that bromocriptine and SKF81297 treatment inhibits lipolysis in rodents [46–48]. Moreover, in subjects with and without T2D, bromocriptine treatment lowers triglycerides, FFAs, and cardiometabolic risk [8, 49, 50]. Such effects to inhibit lipolysis in adipocytes are likely to lead to improvements in circulating lipids with a protective role in the liver and heart [8, 51]. It should also be noted that previous studies have shown contradictory results regarding the involvement of dopaminergic signaling in lipid metabolism. It has been shown that 24-hour incubation of adipocytes from a murine cell line with a specific DRD1 receptor agonist stimulates lipolysis [17]. In another study, bromocriptine treatment in patients induces an increase in circulating FFA levels [49]. Taken together, these data suggest that the effects of dopaminergic signaling might be dependent on different factors, such as duration of treatment, genomic effects [37], communication with other organs, such as the brain or gut, and time-of-day dependent effects [52].

This study of course has some limitations. The correlations are hypothesis-generating and explorative, and they do not assume causality. Thus, functional assessment of dopaminergic signaling on human adipose tissue glucose and lipid metabolism was performed, but in a limited number of subjects and only in SAT. Further, the number of samples used for mRNA analyses was much higher than for protein, and it would have been preferable to analyze protein levels in the same number of samples as we did mRNA expression. Dopamine levels in human adipose tissue are not known, therefore the dopamine concentrations used reflect circulating dopamine levels [26, 27]. Also, in previous publications, dopamine shows a non-linear responsiveness in adipocytes [15] hence, the dopamine concentrations used in this adipose tissue ex vivo approach do not necessarily reflect the in vivo setting. Thus, additional in vivo functional assessment would be preferred, for example in subjects exposed to compounds targeting dopamine receptors.

In summary, DRD2 mRNA and protein expression in SAT was associated with hyperglycemia independently of obesity status and increased in subjects with impaired fasting glucose and T2D. *DRD1* gene expression was increased in subjects with overweight/obesity, but protein expression did not follow gene expression data. Dopamine (1, 50, and 100 nM) did not affect subcutaneous adipocyte glucose uptake and lipolysis ex vivo, but DRD1 and DRD2 agonists inhibited isoproterenol-stimulated lipolysis.

In conclusion, these results suggest that the protein expression of DRD2 in SAT is up-regulated with hyperglycemia and T2D. Whether these changes have direct consequences for the development of T2D, or result from a metabolic adaptation needs further investigation. Additionally, dopamine receptor agonists attenuate adipocyte beta-adrenergic stimulation of lipolysis, which may potentially contribute to beneficial effects in lipid metabolism observed in patients taking bromocriptine.

### Supplementary information

Online Resource 1

## Abbreviations

BMI: Body mass index
CNS: Central nervous system
DRD1: Dopamine receptor D1
DRD2: Dopamine receptor D2
DRD4: Dopamine receptor D4
HbA1c: Glycated hemoglobin
HDL: High-density lipoprotein
HOMA-IR: Homeostatic model assessment of insulin resistance index
ISO: Isoproterenol
LDL: Low-density lipoprotein
OAT: Omental adipose tissue
RYGB: Roux-en-Y gastric bypass
SAT: Subcutaneous adipose tissue
SVF: Stromal vascular cells
T2D: Type 2 diabetes
WHR: Waist to hip ratio
