# Supplementary material for: Subcutaneous adipose tissue dopamine D2 receptor is increased in prediabetes and T2D
Source: Endocrine. 2023 Sep 26;83(2):378–91. doi: 10.1007/s12020-023-03525-1 (PMC10850013; doi:10.1007/s12020-023-03525-1)
Supplement: Supplementary file 1 — Online Resource 1 [file 12020_2023_3525_MOESM1_ESM.docx]

**Online Resource 1**


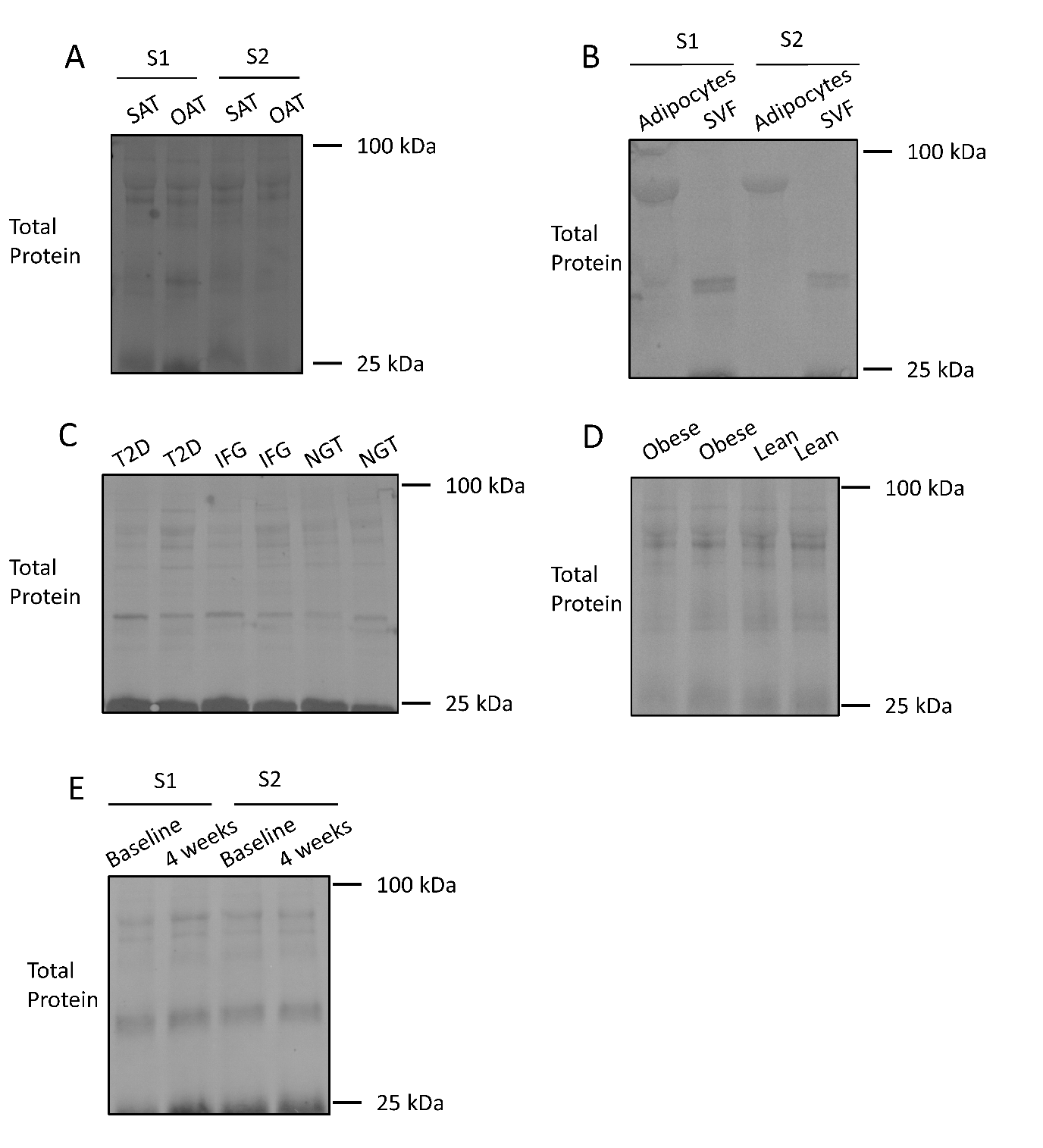


Figure legend. Representative immunoblot of total protein levels. Paired samples of (A) SAT and OAT and (B) adipocytes and SVF; (C) subjects with NGT, IFG, and T2D; (D) subjects with or without obesity; (E) baseline and 4-weeks post-bariatric surgery (RYGB). n = 2. SAT, subcutaneous adipose tissue; OAT, omental adipose tissue; SVF, stromal vascular fraction; NGT, normal glucose tolerance; IFG, impaired fasting glucose; T2D, type 2 diabetes.
